# 

**Published:** 1899-12-30

**Authors:** 


					TBM HOSPITAL. "The standard of highest p?rity."?Lancet. December 30th, 1899.
m i M * mj. 1 UC ai^iuaiu W. y m*m m+j ?        "
CADBURY'S rf H It* CADBURY'S
cogoa % ^ m <m5~> cocoa
ABSOLUTELY PURE. WO DRUGS OR ALKALIE8 USED.
Wlascoiy Western Infirmary ==?-^ 'NojulolKama NORWICH ,
y rxTTT r~T?r?r?iofoi-o/l 0o"1 *-+ ^ rSnTicnrmfinn Torma ~1
No. G92. Vol. XXVII. Saturday,
Dec. 30, 1899.
^Subscription Terms,J pRICE TWOPENCE.

				

